# Review: Resin Composite Filling

**DOI:** 10.3390/ma3021228

**Published:** 2010-02-19

**Authors:** Keith H. S. Chan, Yanjie Mai, Harry Kim, Keith C. T. Tong, Desmond Ng, Jimmy C. M. Hsiao

**Affiliations:** 1 Department of Zoology, University of British Columbia, Vancouver, British Columbia, V6T 1Z4, Canada; 2 School of Dentistry, University of Queensland, Brisbane, Queensland 4000, Australia; E-Mails: yanjie.mai@uqconect.edu.au (Y.M.); juram.kim@uqconnect.edu.au (H.K.); keith.tong@uqconnect.edu.au (K.C.T.T.); desmond.ng@uqconnect.edu.au (D.N.); jimmy.hsiao@uqconnect.edu.au (J.C.M.H.)

**Keywords:** restorations, resin composite, amalgam, history, clinical procedures, finishing and polishing technique

## Abstract

The leading cause of oral pain and tooth loss is from caries and their treatment include restoration using amalgam, resin, porcelain and gold, endodontic therapy and extraction. Resin composite restorations have grown popular over the last half a century because it can take shades more similar to enamel. Here, we discuss the history and use of resin, comparison between amalgam and resin, clinical procedures involved and finishing and polishing techniques for resin restoration. Although resin composite has aesthetic advantages over amalgam, one of the major disadvantage include polymerization shrinkage and future research is needed on reaction kinetics and viscoelastic behaviour to minimize shrinkage stress.

## 1. Introduction

For centuries, the main source of sugar was honey because the sugar from sugar cane in India was expensive and the transition from the use of honey to sugar cane happened during the Industrial Revolution with an increase in the incidence of dental caries [1]. Sucrose is implicated in caries progression because it is a disaccharide and serves as a substrate for extracellular polysaccharide production [2,3]. It is broken down into fructose and sucrose and forms an important structural component of the dental plaque, as well as serving as a metabolic substrate for micro-organisms [2,3]. It has been shown extensively that it is not the quantity of sugar consumed which causes caries but rather the frequency [4]. Caries is a very common disease throughout the world and is the leading cause of oral pain and tooth loss [5]. 

This multifactorial disease is associated with the flow and composition of saliva, amount of fluoride, sugar intake and prevention and can be reversible at an early stage [6]. The progression of the disease is rather slow and can be observed either on the crown or root and pitted and fissured surfaces. The equilibrium between the micro-organisms of the oral cavity and tooth minerals has become disturbed [7]. The bacteria for cavitation are largely dominated by mutans streptococci and *Lactobacillus* spp. and lactic acid is produced from the fermentable carbohydrate [8]. The drop in the pH value promotes demineralization and cavitation occurs when the diffusion of calcium, phosphate and carbonate out of the tooth does not stop [9]. 

Treatment of dental caries can include restoration using amalgam, resin, porcelain and gold, endodontic therapy, removal of decayed dental tissues and extraction. The material resin is of considerable interest in the restoration of caries because this relatively novel method (discovered in the 1930’s) can take for a greater range of shades which is more similar to enamel allowing for the restoration to look imperceptible due to adequate shade matching. It is characterized by a high compressive strength relative to most other restorative materials [10]. It also has tensile strength which is enhanced through the addition of heat. In addition, slight changes in the properties of the resin can provide a wider range of uses (e.g. filler particles, quality of coupling agents, activation process). Here, we provide a brief review of the history and use of resin in dentistry, comparison between amalgam and resin, clinical procedures involved, finishing and polishing techniques and possible future trends for resin restoration. 

## 2. History and Use of Resin in Dentistry

Historically, prior to the 1900’s, there was a rise in the use of plastic-type materials for denture bases to provide an inexpensive denture base. Vulcanisation was discovered in 1839 by Dr Charles Goodyear, Jr., and this process involved the mixing of sulphur with natural rubber for altering properties such as flexibility and hardness; this was followed by the invention of Vulcanite by Nelson Goodyear in 1851 which involves a manufacturing process for vulcanized rubber and the first denture constructed from Vulcanite was created in 1853 [10]. However, although by 1891 the use of Vulcanite as a denture base material was widely acknowledged, another material which is easier to manipulate was required for restorative purposes.

In 1936, the method of using poly(methylmethacrylate) (PMMA) in the fabrication of dentures was first put in practical use [10]. This method opened up a world of possibilities in restorative dentistry as acrylic resin (*i.e.*, PMMA) can be tinted, shaped, injection-moulded and compression-moulded. However, the first forms of PMMA used in denture bases required to be heat-processed. Soon after, a new technology, auto-polymerizing acrylic resin, was released after further research. They are able to set by themselves without additional application of heat, but auto-cured acrylic resin begins to set as soon as it is mixed, thus causing heavy restrictions on the time available for manipulation. In response, again a new technology was developed called light-cured acrylic resin. It requires a device using light that will initiate the curing reaction, such as blue light from a filtered halogen lamp, argon-ion lasers, plasma arc units or light emitting diodes in order to set [10]. Unlike the auto-cured acrylic resin which begins to set immediately after mixing, light-cured acrylic resin has a much longer working time between its application and its setting. Using the same polymerisation principles, resin composite (Bisphenol-A Glycidyl Dimethamethacrylate, Bis-GMA or Bowen’s resin) was finally developed and is used in restorative procedures whereas acrylic resin is used in applications such as denture bases [10]. After the development of resin composite, other restorative materials continued to be developed as no one material is capable of successfully restoring all dental conditions in every situation. Examples include mixtures of resin composite and glass ionomers such as polyacid modified composites (which has a higher fraction of resin composite and lesser amount of glass ionomer—GIC added to resin composite) and resin modified glass ionomer cement (which has a higher fraction of glass ionomer and lesser amount of resin composite – resin composite added to GIC) [11,12].

There are several properties of resin composite that makes it a more favourable restorative material than many others. Resin composite is comprised of several components: an organic resin polymer matrix, inorganic filler particles, silane coupling agent, initiators/accelerators and pigments. One of the most important is resin composites’ highly aesthetic properties as they come in a wide range of shades similar to enamel, determined by the pigments used, thus allowing near invisible restorations of the teeth. In the case of anterior restorations, the shades of resin composite must be judged precisely as it can easily be seen [13]. The type of inorganic filler particles and the ratio between the filler particles and the organic matrix determine the resin composite’s ability to withstand wear and stress. There are essentially two types of filler particles: microfil particles and macrofil particles while a combination of microfil and macrofil particles are termed “hybrids” [14]. Whereas macrofil particles are reserve more strength, larger fractions of microfil particles are more often used for the anterior teeth as resin composite comprising of more microfil particles tend to be easier to polish, allowing for a much more aesthetic finish [14]. Similarly, by increasing the proportion of macrofil particles, resin composite can be made to withstand larger compressive forces in the mouth and is commonly used in posterior restorations as their main functions are to grind and crush [15]. This provides a higher compressive strength than most restorative materials with the exception of amalgam [16]. Furthermore, use of light-cured resin composite provides dentists with longer working time as it only sets when the blue light is applied. The longevity of restorations using resin composite is approximately 7 years, which is longer than most other restorative materials with the exception of amalgam, which can last for over 10 years [17]. Nanofilled resin composites contain nano-fillers which form clusters called nano-clusters. Essentially these clusters are similar to micro-fillers and can be very well polished, but can act similar to a large particle providing strength and tend to have less shrinkage [18,19]. 

Properties of resin composite can be altered to suit a wider range of uses by modifying several factors such as the size of the filler particles, quality of coupling agents and the activation process. Depending on these properties, resin composite can be used at different parts of the oral cavity. As mentioned, resin composite containing only microfil particles or a higher fraction of microfil particles (called a hybrid) are generally used in the anterior teeth due to its high polishability [14]. Resin composite with higher tensile strength due to a larger proportion of macrofil particles are generally used in posterior teeth and, based on basic material science principles for brittle-like materials (e.g., glassy rein composites), tensile strength properties are more relevant than compressive strength properties when comparing other restorative material types. Resin without the filler particles tend to be more flowable and are thus used as pit and fissure sealants [15]. Nano-filled resin composite, due to its polishability, strength and reduced shrinkage, are frequently used for larger, posterior restorations [18,19].

## 3. Amalgam and Resin

Amalgam has been used as a dental restorative material since the 19^th^ century, over 150 years ago, and has been used as the preferred material for nearly all restorations not requiring high levels of aesthetics [20,21]. The development of early resin composites in the late 1950s and early 1960s led to the introduction of a resin composite material as a class II restorative in 1968 leading to a constant decline in the number of amalgam restorations [22,23]. Between the periods of 1979 and 1999, the number of amalgam restorations placed in the United States had decreased from 157 million to 66 million, and the relative number of resin composite restorations placed exceeded that of amalgam based materials [20]. In current dental practice, resin composite is used for anterior restorations as well as posterior restorations, generally Class I and Class II restorations [24].

Modern dental amalgam is composed of mercury, silver, tin and copper which is an alloy with a dull metallic colour, not matching well aesthetically with the natural teeth [20]. The development of resin composite presents the benefit of aesthetics, being able to be modified to match the colour and translucency of the patient’s teeth [20,25]. In the past, the common opinion on restorations was focused on its longevity. Moreover, one reason why amalgam remains a popular form of treatment is due because it is much easier to place than a resin composite. However, while the dentist is still concerned on longevity and technique sensitivity, patients are concerned about aesthetics [26]. The public opinion on restorations has changed significantly, patients are now aware that the aesthetic alternatives are also possible and metal restorations are not necessary [27]. It has been shown in the United States and the United Kingdom that there is patient interest in tooth-coloured restorations [28], and patients request tooth coloured restorations, often not informed about the availability and quality of metal restorations [27]. Currently, the ability of resin composite to imitate the colour, transparency and anatomical form of surrounding teeth through correct placement has given it enhanced aesthetic value [29], it is used in 50% of all posterior direct restorations [25].

Resin composite restorations are not only more aesthetically pleasing but may also offer clinical advantages over amalgam. While dental amalgam has been used for Class I and Class II restorations successfully for over one and a half centuries, it has been shown that there are shortcomings which can be overcome with resin composite. One of the major advantages of using resin composite as a restorative material is the ability of resin composite to bond with enamel, unlike amalgam. This micromechanical retention is shown to be simple to develop, and is the strongest adhesion in the oral cavity currently available [29]. As a result, there is less tooth structure loss in the cavity preparation when compared to the placement of a similar amalgam restoration where a retentive form may be required in order to keep the amalgam in the preparation. Furthermore, it has been shown that there is strengthening of the tooth from the resin composite restoration, with amalgam restored molars showing less stress than an unrestored molar and resin composite restored molars showing less stress than an amalgam restored molar [30]. Resin composite has also been shown to have low cusp fracture rates of 2.29%, not significantly different from that of amalgam at 1.88% [31]. Another factor which shows the efficacy of resin composite as a material is the amount of wear subjected. Amalgam has always been shown to have a lower rate of wear compared to resin composite in the past [20]. However, recent technology of resin composite has improved and current resin composite technologies have wear rates of 110–149 micrometres after 3 years, very similar to those of human enamel structure at 122 micrometres after 3 years [24,32].

The longevity of the resin composite restorations has been in question for many years. In comparison, the long history of amalgam restorations has proven the longevity of amalgam restorations. Amalgam is known to have long median survival times of 55 to 70 years under skilled dentists with few time constraints [33]; however, in normal practice, it is observed that amalgam typically has an expected longevity of at least 10 years [23]. The longevity of earlier resin composites was comparatively shorter, with higher failure rates, with data showing a median survival time of 3.3 to 4.7 years compared to 6.6 to 14 years for amalgam [33]. However, observations from normal practice show that most dentists can place resin composites which have been shown to serve at least 10 years [23]. On the other hand, some of the trials have found that resin composite and amalgam have similar survival rates, with resin composite at 91.7% at 5 years and 82.2% at 10 years and amalgam at 89.6% at 5 years and 79.2% at 10 years [33]. This is consistent with another study which shows that the yearly failure rate for resin composite was 0 to 9% compared to that of amalgam at 0 to 7% [26]. The current evidence shows that while amalgam has a proven track record as a long lasting restorative material, the current technologies of resin composite also shows promising results of a future restorative material. 

Amalgam has long caused much concern of its effects to systemic health due to its mercury component. Studies have shown that minute quantities of mercury vapour may be released from amalgam restorations in the process of mastication [20,21]. The inhaled mercury vapors are then absorbed by the lungs, where it diffuses and enters the blood [21]. As a result, there have been several examples of regulations and recommendations set in differing countries to limit its use due to health concerns and environmental reasons [28]. Some of the governmental restrictions in such countries include the recommendation of avoiding amalgam restorations in pregnant women and young children, and the limitation of amalgam restorations on molars only [21,28]. It has been abandoned or it is strongly discouraged as a restorative material in clinical use in some countries (e.g., Scandinavia) today. There have also been concerns in the past on resin composite and the presence of bisphenol A; however, they have been resolved and studies show that resin composite, like all restorative materials, have negligible levels of toxicity [20]. More realistic problems likely to occur in practice are based on the biocompatibility and post operative sensitivity of the materials. While there are very little cases of amalgam and resin composite sensitivity, both cases do occur rarely [20]. Another factor to be considered is that approximately 60% of the amalgam waste removed from teeth when replacing restorations escapes into waste waters [29]. This is a concern as the waste waters can enter the food chain from the waterways, affecting people who then consume animal products such as fish [29]. Resin composite may not only be a safer material for restorations but also have less effect on the environment.

## 4. Clinical Procedures 

The technological advances and evolution in restorative dental material has made resin composite to be the material of choice worldwide for Class II restorations. Its ability to bind tooth structure readily with adhesives and most importantly closely matching the shade of natural teeth has led to its high utility among clinicians [34,35,36,37,38,39]. With proper clinical procedures and technique taken by the clinicians, this material can achieve predictable and successful results. Direct resin composite restorations require a technique delicate approach. Limitations to this technique may arise due to sensitivity of the material in the mouth, insufficient binding to dentine and polymerization stresses and shrinkage during light-curing [40,41]. These problems are more prevalent in posterior teeth as it is difficult to achieve isolation in these areas. Thus it is important that the clinician consider the following factors during direct resin composite restoration (1) proper isolation of teeth; (2) sound tooth structure must be preserved; and (3) prevention and treatment of dental caries and periodontal diseases have been looked into before commencing the restoration process [34].

**Figure 1:**
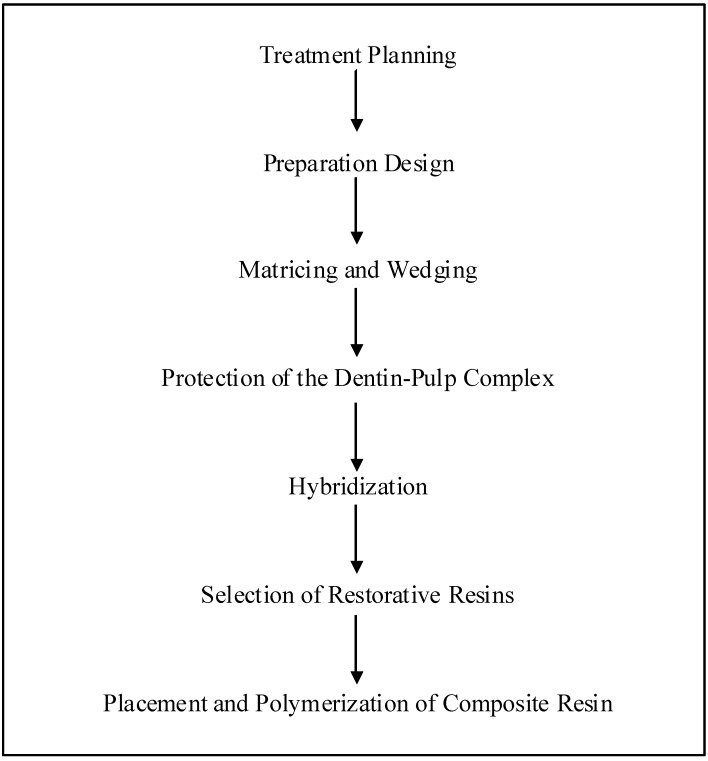
Clinical procedures of a direct resin composite restoration.

Before the commencement of the resin composite restoration, the extent of the lesion must be thoroughly examined through the use of bitewing and periapical radiographs [34]. This would help the clinician in forming the restoration plan (Figure 1). Vitality tests, prophylaxis, occlusal analysis, selection of tooth shades and tooth separation are necessary procedures that have to be taken before the commencement of cavity preparation [42,43].

Preparation is restricted to the area of the cavity (Figure 1). It is often necessary to enlarge the access to dentinal caries to have better instrumentation and visualization; after local anesthesia is delivered, the cavity preparation is made with a round carbide bur in a high-speed handpiece [34]. To remove infected dentine, an inverted cone with round end bur is used at low speed with the size being compatible to the lesion’s extension, together with a spoon excavator and unsupported enamel can be retained if it does not affect the placement of matrices and polymerization of the restorative material. Rubber dam is used to isolate the restoration site and aid moisture control. Once preparation is completed, matrix and wedge are positioned in place.

It is necessary for the clinician to select and install the matrices for the restoration of proximal boxes with resin composite (Figure 1) [34]. Thin pre-contoured metallic matrices are preferred as they would be able to achieve a proper contour and allow for interproximal contacts without overhangs. The placement of matrices will depend on the bucco-lingual extension of the proximal box. An alternative method would be to use thin precontoured metallic matrices together with a ring to hold the position of the matrix as well as to create a slight gap between the tooth being restored and the adjacent tooth [44].

Glass-ionomer cements are currently advocated as base materials for these restorations to act as dentin adhesive material, promoting the adhesion between natural tooth and resin composite [45]. However the weak bond strength and its handling properties have made it a less popular choice among clinicians. Hybridization of the exposed dentin with an adhesive system provides adequate protection of the pulp-dentin complex under resin composite restorations (Figure 1) [46,47,48,49].

The use of contemporary polymer adhesive systems is mainly to protect the dentin-pulp complex as well as to bond the restorative resin composite to the restorative resin composite to the natural tooth, preventing microleakage [40]. Acid-etching of enamel and dentin is used widely in procedures of dentin bonding [34]. The total etch technique relies on the micro-mechanical retention established by the acidic treatment of enamel and dentin prepared surfaces and successive infiltration of a blend of polymers into the etched substrate [50]. Bonding to dentin is more difficult than enamel due to the tubular structure of dentin and high-vapor-pressure organic solvents are currently used to carry resin monomers into contact with dentin collagen [34]. The application of acidic etchant is to be applied continuously to the enamel and dentin and the dentin layer is to be kept slightly moist after the removal of the acid etchant using water. When the etchant has been removed and the surface slightly dried, the adhesive system is applied. The dentin adhesives used by clinicians depend on the permeation of hydrophilic monomers into acid-conditioned dentin, resulting in the resin being strongly associated with collagen and hydroxyapatite crystals which is known as the “hybrid layer” [50]. This layer is known to give higher binding strength, especially when acid-etched dentin is in a partially moist condition [51,52].

Research has shown that the most suitable resin composite for use would be the hybrid bimodal small-particle light-cured composites or “microhybrids” [53]. These resins have an average particle size of 0.6 µm to 0.8 µm and contain a microfiller with a size of 0.04 µm and the microhybrids contain up to 78% by weight of filler, giving the resins superior physical and mechanical properties which would be useful in the posterior quadrant [34]. Examples of microhybrids include Herculite XVR, Prodigy, Kerr/Sybron, Orange, CA; Charisma, Heraeus Kulzer, South Bend, IN; Pertac II, ESPE, Norristown, PA; TPH-Spectru, Dentsply/ Caulk, Milford, DE; Glacier, SDI, San Francisco, CA [25].

Research has proven that the most effective placement technique to be horizontal, oblique, vertical and bulk incremental technique (Figure 1) [54,55,56,57]. The incremental technique has been shown to keep polymerization shrinkage to the minimum, provides improved control of “condensation” of the individual increments of resin composite, thorough polymerization of the restorative material, and limit overhangs in the lateral margin subsequent to curing [34]. This technique also allows for the control of the contour on the occlusal surface of the restoration during the final stages of increments. The number of increments applied varies proportionally to the size of the cavity and the application of resin composite should be guided according to the anatomy of the tooth. Light-cured composites pose an advantage in that clinicians are able to apply and shape the resin composite to the final contour during the setting of the material. Uncured composite can be shaped with different metal or plastic instruments to achieve contour and smoothnesss. Despite the advantages posed by this technique, it however demands a high level of dexterity from the clinician.

## 5. Finishing and Polishing Techniques for Resin Restorations

Finishing and polishing can be thought of as two steps of a same process. Finishing corrects contours while margins and irregularities are smoothed by abrasion [58]. Simply put, finishing removes excess material and adjusts occlusion while final polishing uses extremely fine abrasives in order to minimize the remaining roughness [59]. Polishing works to produce a smooth surface in order to prevent retention of plaque which is a major initiating agent of caries and periodontitis [58]. Diamond rotatory cutting instruments, carbide burs, abrasive-impregnated rubber cups and points, abrasive discs, and polishing pastes are some of the typical products used in finishing and polishing [60]. 

Prior to finishing and polishing, several procedures are undertaken to ensure the success of restoration. First, there is an evaluation of restorations for detection of defects such as chips, stains, and voids [61,62]. The surfaces of tooth should be dried before evaluation as composite restoration and natural tooth structure share similar colors. The restoration may be examined visually or by tactile conduction with an explorer on margins and proximal surfaces. Adjunctively, a dental floss may be used to verify proper contacts and gingival margins [61]. 

After the examination, the clinician or dentist discusses the procedure with the patient. Patients are informed about the rationale and procedures of finishing and polishing, as well as sensations once may experience [61]. In the final step of preparation, rubber dam and cotton rolls are placed for isolation, moist control. Interestingly, this step is rather important as their usage have been found to influence both initial quality and later survival of composite restorations [63]. For aesthetic restorations, it is important to determine which finishing system is most suitable for a specific restoration since different composites differ in their ability to be polished, mostly due to variations in their inorganic component [58]. Table 1 lists the surface roughness of resin composites from different types of finishers and polishers. 

**Table 1:** Representative surface roughness (μm) of composites finished with various abrasives [58].

Finisher/ Polisher	Multipurpose Composites	Microfilled Composites	Packable Composites	Laboratory Composites
Mylar Surface	0.05	0.06	0.13	0.03
Diamond Finishing Bur	1.35	N/A	1.60	0.74
Carbide Bur	0.67	0.24	0.63	0.24
Composite Polishers	0.29	0.15	0.37	0.14
Aluminum Oxide	0.12	0.09	0.16	0.09

A curved scalpel blade is often utilized in removing interproximal overhangs and flash [61]. Overhangs, frequently found on proximal surfaces, determine which finishing and polishing procedure should be incorporated. Scalpel blade should be held approximately at acute angle with tooth surface in contact with the cutting edge. Flame shaped finishing burs are also used to remove overhangs. Additionally, flame shaped burs are used to smooth contours and margins. As with disks, it is recommended to move from tooth structure to restoration using smooth, deliberate, intermittent brush strokes (SDIBS). This is done in constant motion while engaging the surface to prevent gouging the restoration or tooth surface.

Finishing disks are useful when the restoration extends onto the facial or lingual surface. Most finishing discs are coated with an aluminum oxide abrasive which is usually disposable or for single-use [64]. Finishing disks should be used with SDIBS movement starting on tooth structure, then over the surface, and remove flash [61]. Lastly, finishing strips without abrasives on its centre are used by pulling or “flossing” the central portion through the proximal contact. In addition to proximal “flossing,” the strip may be positioned on tooth structure and restoration to “floss” margins on restoration. Finishing strips may be used repeatedly if needed. Note that gapped finishing strips are used for interproximal finishing and polishing [64]. 

Occlusal finishing smooth occlusal margins, refine anatomy, and removes flash [61]. Egg or football-shaped finishing burs complete almost all of occlusal finishing. Existing tooth structure is used as a guide for the bur over triangular ridges. As before, SDIBS movement should be maintained throughout the procedure. Finishing points and cups are also used with SDIBS movement to smooth the surface and grooves. Also, rubber or flexible finishers complement coated abrasive disks which have limited access to posterior occlusal and anterior lingual surfaces [64]. 

Smooth margins, refined axial contours, removal of flash, and elimination surface stains are accomplished by finishing facial and lingual surfaces. Flame-shaped finishing burs are used on latch-type contra-angle handpiece to contour surfaces, smooth margins, and remove flash [61,64]. As with other abrasives, SDIBS movement is applied. The instrument should maintain constant motion while engaging the surface to prevent gouging the restoration or tooth surfaces. Afterwards, egg or football-shaped finishing burs are used on the lingual surfaces of anterior teeth with SDIBS movement. Bur should be guided by existent tooth structures. 

Finishing disks and cups are commonly used on facial surfaces of anterior teeth while finishing points and cups are used on lingual surfaces of anterior teeth. For posteriors, a combination of disks, points, and cups is recommended. Also employing SDIBS movement, the procedure should initiate at the tooth structure, and then moved over the restoration surface. At this point, the clinician should assess the finishing and decide if all criteria have been met. One should proceed to polishing only if all criteria are satisfied [61]. 

The process of polishing composite restoration is very similar to that of finishing. However, polishing makes use of relatively fine abrasives, removes less bulk material, and achieves a lustrous, smooth surface [61,65]. On the proximal surface fine abrasive disks and strips or rubber polishing disks, points and cups are utilized during polishing procedure with SDIBS movement. Like finishing, polishing should start from tooth structure, and continued over the surface of restoration to produce a smooth surface with high lusture [61]. 

Polishing points or cups are used in the final polishing procedure on occlusal surfaces. Points and cups are moved across the surface in smooth and deliberate strokes [61]. As for facial and lingual surfaces polishing points, disks, or cups are used during the final polishing procedure. Similarly, instruments are moved across the surface with smooth and deliberate strokes. Finally, the polishing procedure is evaluated with a dental mirror and explorer. The composite restoration should appear smooth and highly polished, and reflect a lustrous shine. Final luster can be obtained by brushing with a goat hair Chamois brush until the polishing paste becomes dry [66]. There should be no damage to the adjacent tooth structure. If polished successfully, the restored surface should yield an appropriate luster and polish [66]. Occlusion should be verified after finishing and polishing [66,67]. 

## 6. Future Trends

Despite the aesthetic improvement of resin composite relative to amalgam, one of the disadvantages of resin composite is shrinkage from polymerization and the factors influencing polymerization shrinkage include volumetric shrinkage and viscoelastic behaviour [68]. Volumetric shrinkage refers to the reduced distance between two categories of atoms when a covalent bond is formed between monomers as well as the reduction in free volume [69]. Three factors contributing to the magnitude of polymerisation shrinkage stress include the resin composite’s composition, conversion of the resin matrix and its filler volume fraction [68]. The value of shrinkage for commonly used resin composite ranges from 2% to 3%, with considerably larger reported value for 2,20-bis-[4-(methacryloxypropoxy)-phenyl]-propane BisGMA (5.2%) and tri-(ethylene glycol) dimethacrylate TEGDMA (12.5%). Resin composite displays viscoelastic deformation when it is prepared with instant load which is applied for a certain amount of time. Different resin composite responds in different ways depending on its filler content, matrix chemistry and degree of conversion [70]. Due to the higher elastic modulus, lower strain capacity and similar volumetric shrinkage of hybrid resin, they develop higher stress value compared to the microfilled resin composites. Similarly, as high cross-linking density deters chain movement in the polymer network and increases polymer chain entanglement, materials with low degrees of conversion experience more deformation. Currently, there is no technique to encounter the problems raised by shrinkage stress except for an understanding of the mechanisms involved in shrinkage stress and this allows dentists to reduce the destructive impacts. Therefore, future research should emphasize on reaction kinetics and viscoelastic behaviour of new dental materials [68]. 

The development of resin based composite (RBC) system highlights a gradual decrease in filler particle size. There has been a significant shift of increased usage of nanofilled or nano-hybrid RBC in recent years [71]. The nanofilled RBC is a combination of dispersed filler particles (5–75 nm) and an incomplete porous cluster (~1.3 µm) of agglomerated particles infiltrate with silane; these are merged into the RBC matrix and are described as nanoclusters. To investigate the influence of nano-sized filler particles and nanoclusters on the strength and reliability of RBC, the influence of dry and wet cyclic preloading on bi-axial flexure strength was determined by comparing seven commercial RBC restoratives containing different size of filler particles [72]. Results indicate nanofilled RBC displayed enhanced reliability in strength and additional resistance to fracture regardless of environmental circumstances. This study also found that when internal porosities of nanoclusters and interstices are infiltrated with silane coupling agent, an interconnected network would be produced, forming an interpenetrating phase composite (IPC) structure. The infiltrated IPC structure has been proved to possess significantly enhanced mechanical property compared with other RBC’s. 

However, certain deficiency associated with nanofilled RBC’s have been discovered [73]. In comparison with traditional fillers, which hold irregular or spheroidal structures, nanofillers were described to display a greater tendency towards multiple fractures. In addition, the relatively higher load prior to and during fracture and higher variability of psedo-modulus properties exhibited by nanofilled RBC can alter the tolerance of the entire system [72]. Therefore, more improvement and research into nanotechnology on nanofilled RBC is required to produce an optimal outcome for future dental practice. 

With development of modern technology RBC materials has not only modified size of fillers, but also its chemical composition. With the aim of low shrinkage, high reactivity and biocompatible RBC that is viable a variety of oral environments, a new cationic ring opening monomer system was developed [74]. Of all the four methacrylate based materials examined in terms of their modulus, flexural strength, compressive strength and ambient light stability, silorane RBC exhibits the lowest polymerization shrinking stress (0.94% of volume by bonded disk method and 0.99% of volume by Archimedes method) [74]. Only one of the methacrylate based materials Tetri Ceram display similar reactivity with silorane composite. Nevertheless, silorane composite showed slightly higher light stability (10 min) compared with other methacrylate based materials which range from 55 to 90 seconds. Therefore, despite slightly longer light stability, the silorane composite exhibits many factors required to be a successful restorative material, including minimizing stress caused by polymerization shrinkage and high reactivity.

Another approach for alternating the chemical composition of RBC includes the investigation of the effectiveness of potentially expanding monomers to reduce polymerization shrinkage stress [75]. The mixture of aliphatic dioxirane and a potential expanding monomer was obtained after photopolymerisation with a dental curing lamp and these were analyzed by NMR to measure to physical properties of polymer solids. By using quantum mechanical calculation, the results reveal that the photopolymerization of a potential expanding monomer and an aliphatic dioxirane exhibits likelihood of significantly reducing polymerization shrinkage stress by making cross-linked copolymer resins. 

## 7. Conclusion

In this article, we have attempted to provide a brief review on the use and benefits of resin for the restoration of dental caries by discussing the history and use of resin in dentistry, comparing the amalgam and resin in the treatment of caries, standard clinical procedures for resin restoration and finishing and polishing techniques for resin restorations. Amalgam had been the preferred material for restoration in the early 1900’s but its use has recently been surpassed by resin composite [20,21]. Arguably, the major reason for the substantial increase in resin composite is its ability to be matched to the colour and translucency of the patient’s teeth [20,25]. Given that amalgam has a longer lifespan relative to resin composite, it is likely that the patients care more for aesthetics rather than the length of time their restorative materials last. In addition to these benefits, resin composite has also been shown to offer clinical advantages such as enamel bonding and it helps to strengthen the tooth [30]. 

While resin composite shows several features of a good dental restorative material, there are many shortcomings and challenges for the resin composite technology to overcome. In terms of aesthetics, resin composite being a tooth coloured material is much more pleasing for the patient and in clinical performance, resin composite is shown to be close to matching the properties of amalgam which has allowed it to be in use for the past 150 years. However, while the longevity of resin composite is about 7 years, amalgam typically lasts longer [17]. It is important to note that resin composite is showing a strong future with its progress in its technology, as well as following the principles of minimal intervention dentistry. On the other hand, it must be noted that despite its progress in recent years, resin composite still has its shortcomings in terms of more heavily loaded and longer serving restorations, and amalgam should still be utilised in these cases. The main emphasis in dentistry currently are aesthetics and minimal intervention and thus, resin composite should be developed as the major restorative material able to be used safely in most situations, and able to serve patients for an acceptable length of time.
